# Influence of Sepiolite and Lignin as Potential Synergists on Flame Retardant Systems in Polylactide (PLA) and Polyurethane Elastomer (PUE)

**DOI:** 10.3390/ma13112450

**Published:** 2020-05-28

**Authors:** Valentin Carretier, Julien Delcroix, Monica Francesca Pucci, Pierre Rublon, José-Marie Lopez-Cuesta

**Affiliations:** 1 Centre des Matériaux des Mines d’Alès (C2MA), IMT Mines Alès, Université de Montpellier, 6 Avenue de Clavières, 30319 Alès Cedex, France; Julien.Delcroix@mines-ales.fr (J.D.); monica.pucci@mines-ales.fr (M.F.P.); jose-marie.lopez-cuesta@mines-ales.fr (J.-M.L.-C.); 2 Centre d’expertise des Structures et Matériaux Navals (CESMAN), Naval Group Research, Technocampus Océan, 6 rue de l’Halbrane, 44340 Bouguenais, France; pierre.rublon@naval-group.com

**Keywords:** fire retardants, PLA, PUE, flame retardancy, lignin, sepiolite, APP

## Abstract

A comparison of the influence of sepiolite and lignin as potential synergists for fire retardant (FR) systems based on ammonium polyphosphate (APP) has been carried out in polyurethane elastomer and polylactide. Different ratios of kraft lignin and sepiolite were tested in combination with APP in both polymers. The thermal stability and the fire behavior of the corresponding composites were evaluated using Thermogravimetric Analysis (TGA), a Pyrolysis Combustion Flow Calorimeter (PCFC) and Cone Calorimeter (CC). The mechanisms of flame retardancy imparted by APP and other components were investigated. Synergistic effects were highlighted but only for specific ratios between APP and sepiolite in polyurethane elastomer (PUE) and polylactide (PLA) on one hand, and between APP and lignin in PLA on the other hand. Sepiolite acts as char reinforcement but through the formation of new phosphorus compounds it is also able to form a protective layer. Conversely, only complementary effects on fire performance were noted for lignin in PUE due to a dramatic influence on thermal stability despite its action on char formation.

## 1. Introduction

For decades, polymeric materials have been used in a plethora of application fields. Some of these fields are directly concerned with fire regulations. Indeed, the inherent flammability of polymers is one of the main issues for the use of this kind of material. In order to improve the fire behavior, the polymer industry is working on materials containing flame retardants (FR) to obtain formulations exhibiting low flammability. Various systems, reported in several reviews [1,2,3], have been used to improve the fire reaction of the different types of polymers and containing mainly phosphorus compounds [4,5,6,7], but also organo-modified layered silicates (OMLS) [8,9,10] and more recently biobased fire retardants [11,12,13]. This wide variety of systems reflects the complexity to find an efficient combination for each kind of polymer. For example, some flame retardants like phosphorus ones are particularly devoted to oxygen and nitrogen containing polymers but they are not relevant for polyolefins.

Poly(lactide) (PLA) is a biobased aliphatic polyester obtained from agricultural plants which is also renewable and biodegradable [14]. PLA is a thermoplastic well-used in food-packaging technologies [15] and in biomedical engineering [16]. The increasing use of this polymer is due to its eco-friendly and biocompatible character. It is one of the most used biopolymers [17] and is easily processed because of its good melt properties. However, PLA has a low toughness, ductility and a poor thermal stability [18]. Although PLA is mainly used in packaging, the production of durable goods in PLA is developing, for example in the areas of toys or domestic appliances. Moreover, PLA has an important potential in the field of additive manufacturing and especially for processing by FDM (Fused Deposit Modeling) [19]. Therefore, flame retarded PLA needs to be developed in order to meet fire safety standards for uses in applications where fire regulations operate.

Polyurethanes are also commonly used in a number of applications often requiring flame retardancy. Segmented ones, consisting of soft and hard segments, are used for various applications such as coatings [20], seals [21] or shape memory materials [22]. As an engineering polymer, polyurethane elastomer (PUE) has been widely used in industrial sectors like aerospace, medicine, automobile manufacturing or shockproof buffer materials due to its high performance in terms of excellent abrasion resistance, chemical resistance and high elastic properties [23,24,25]. PUE can also be injected, molded or extruded. However, the high flammability of polyurethane with melt dripping [26,27,28] is a major limitation of this material. Improving the flame retardancy of PUE is then an important challenge to extend its applications. Recently, the flame retardant properties of PUE have been improved using different kinds of additives [29,30,31,32,33].

In this context, intumescent fire retardant (FR) systems based on ammonium polyphosphate (APP) have been developed for many years and have been shown to improve the fire performance of both polymers [34,35,36,37]. Poly(lactide) and thermoplastic polyurethane have been selected and compared as polymer matrices with the same additives in this study also because only polyurethane contains nitrogen in its chemical composition, which can modify the reactivity towards the different FR systems used.

It was reported that these FR systems could play a role in both condensed and gas phases [38]. They could reduce the fraction of flammable materials and help in forming a protective char due to a strong dehydrating agent generated by polyphosphoric acid. This char is a residue containing phosphorus which can limit oxygen access to prevent the spread of flammable gases and carbon gasification [39,40]. However, a composite with only APP as a flame retardant cannot effectively yield enough fire performance. Hence, it is necessary to combine APP with other flame retardants to achieve synergistic effects on flame retardancy [41,42]. In the last 20 years, polymer nanocomposites have attracted much interest. Nanoparticles, such as organomodified layered silicates (OMLS) or carbon nanotubes were used successfully to improve fire reaction properties such as heat release rate, by decreasing the peak heat release rate (pHRR), reinforcing the char residues or reducing the mass loss rate [43,44,45]. Only a few works have reported the use of sepiolite in flame retardant systems, in particular with APP [46,47]. This filler belongs to the structural family of 2:1 phyllosilicates with the following formula (Si_12_Mg_8_O_30_)(OH)_4_(OH_2_)_4_·8H_2_O [48,49]. Sepiolite exhibits a microfibrous morphology with tunnels that grow up in the fiber direction. During the combustion, it has been shown by Lewin et al. [50] that OMLS could migrate to the surface and consequently, it can be expected that sepiolite could also behave similarly, reinforcing the char layer. Moreover, regarding other kinds of nanoparticles, recent studies have shown that silica could accumulate on the surface during material burning and lead to a significant effect on the reduction of heat release rate of a polymer [51,52].

New biobased fire retardants are emerging in order to lead to eco-friendlier composite materials. Lignin is one of the more promising biobased components of fire retardants systems [53]. It is a byproduct of the paper industry [54]. Lignin is also a biobased copolymer mainly composed of phenolic groups. The basic units are coumaryl, coniferyl and sinapyl alcohols [55]. Lignin has a good thermal stability and contributes to flame retardancy in composite materials by promoting the formation of char [37,56,57,58]. Few papers have been published on the fire behavior of composites and polymer blends containing lignin. Ferry et al. [56] used unmodified and modified lignin modification in order to improve PBS (polybutylene succinate) fire behavior. Other studies have explored the effect of lignin on the thermal properties of polymers such as poly(ethylene terephtalate) [59] and for polypropylene [60]. Lignin has also been studied as an additive in biopolymers, like in polyhydroxybutyrate (PHB) [61,62].

This work aims to compare the effectiveness of sepiolite and lignin as potential FR synergistic agents of APP in PLA and PUE. The interest of such compounds was investigated for different mass ratios with APP ranging from 1.5 to 5.67, APP being always the major component. A global loading of 20 wt% of FR components was selected in accordance with the loadings of intumescent FR currently used in research about the fire retardancy of these polymers. The greatest attention was focused on the mechanisms of fire retardancy induced by the presence of both additives in each polymer as well as on their influence on thermal stability. Fire behavior was assessed either at the microscopic scale using combustion microcalorimetry or at the macroscopic scale using cone calorimeter. Moreover, investigations on cone calorimeter residues were carried out to attempt to account for the interactions occurring between the different components during thermal degradation.

## 2. Materials and Methods

### 2.1. Polymers and FR Components

The poly(lactide) (PLA) used in this study was supplied by NatureWorks (Minnonka, MN, USA). The polymer is the grade 3251D with a glass transition temperature (Tg) of 55–60 °C, a melt temperature (Tm) of 188–210 °C and a density of 1.24 g/cm^3^. Polyurethane, supplied from Courbis (Romans-sur-Isère, France), was prepared from polytetramethylene ether glycol (Mn ≈ 2000 g·mol^−1^), pre-polymer terminated 4,4′-diphenylmethane diisocyanate (pMDI) and 1,4 butanediol (BDO) as chains extender. APP (Exolit AP423) was provided from Clariant (Muttenz, Swiss). Its phosphorus percentage is 31 wt% and the median diameter of particles is 9 µm. Sepiolite (S9 Pangel, Tolsa Company, Madrid, Spain) was purchased from Lavollée S.A. (Levallois-Paris, France). The selected lignin is a commercial low-sulfonated alkaline lignin supplied by Sigma ALDRICH (Darmstadt, Germany). The median diameter of lignin particle is around 50 µm. All chemicals were used without any purification.

### 2.2. Sample Preparation

The PLA composites were prepared using a twin-screw extruder (1200 mm) Clextral (Firminy, France). The process conditions were the same for all samples. The temperature profile of the barrel was from 80 to 210 °C and the screw rotational speed was set at 250 rpm. The molding process was carried out using an injection molding press (Krauss-Maffei, Munich, Germany) with a closing strength of 50 tons. The blends were injection-molded into plates (100 × 100 × 4 mm^3^). The temperature profile was 40 °C for the mold and 200 °C for the screw.

For PUE, polyol (68.6 g) and pMDI (106.2 g) were held at room temperature 12 h before vacuum drying at 80 °C for 6 h in a vacuum oven. Then, polyol and pMDI were placed in a plastic beaker and stirred for 5 min, after that 9.2 g of BDO was added. The mixture was then cast in a metallic mold heated at 115 °C to obtain four square sheets of size 100 × 100 × 4 mm^3^. The mold was maintained at 115 °C for 1 h 30 min, after which the samples were placed at 115 °C for 15 h. To obtain PUE composites, fillers were added with polyol and pMDI.

Table 1 shows all the formulations and the components ratios for the different compounds. A total amount of 20 wt% for the additions was kept constant. It should be noted that a complete substitution of APP in polymers by each additive was not possible or desirable, leading to processing difficulties or very poor thermal stability due to hydrolytic degradation. Hence, investigations on synergistic effects will be only assessed regarding partial substitutions.

### 2.3. Characterization of Thermal Stability

Thermal stability characterizations of samples were performed using a Thermogravimetric Analysis (TGA) apparatus (SETSYS evolution, Setaram, Caluire, France). Experiments were performed in the temperature range from 30 to 900 °C, at heating rate of 10 °C/min under nitrogen atmosphere (40 mL/min). The sample weight was approximately 12 mg. Initial decomposition temperatures (T_ON-SET_) were determined at 5% of weight loss and the maximum degradation rate temperature was measured from the derivates of thermogravimetric curve (dTG) peak maximum. The weight residue left at 900 °C was determined for all the samples.

Thermal degradation of the different nanocomposites was investigated using a Pyrolysis Combustion Flow Calorimeter (PCFC) (Fire Testing Technology, East Grinstead, UK). PCFC was firstly developed by Lyon and Walters [63] to study the thermal degradation of samples at the microscopic scale. Sample weights were around 5 mg. Samples were pyrolyzed at 1 °C/s, then the volatile thermal degradation products were swept from a pyrolysis chamber by an inert gas and combined with excess oxygen in a tubular furnace at flame temperatures, to force complete combustion. Heat release rate (HRR) was measured as a function of temperature. This enables the determination of the peak value of HRR as well as the Total Heat Release (THR, area below the HRR curve). The results were averaged with an error of less than 5%, while the error on temperature of the peak value of HRR and measurement is less than 1%.

### 2.4. Characterization of Fire Behavior

A Fire Testing Technology cone calorimeter was used to evaluate fire reaction properties. The 100 × 100 × 4 mm^3^ samples were exposed to a radiant heat flux of 50 kW/m^2^. The distance between the cone and the sample was 25 mm. The air flow was 24 L/s. Heat release rate was measured as a function of time. Then, time to ignition (TTI), Total Heat Released (THR), peak of HRR (pHRR), Maximum Average Rate of Heat Emission maximum value of the average of HRR as function of time (MARHE), and quantity of residue were determined.

For each composition three samples were tested and mean values were reported.

### 2.5. Characterization of Microstructures and Cone Calorimeter Residues

The cone calorimeter residues were analyzed by X-Ray Diffraction (XRD) in order to investigate the degradation mechanisms of the various materials studied. The XRD patterns were obtained using an X-ray diffractometer BRUKER D8 Advance. A scanning angle of 2θ was from 5° to 70° with an X-ray beam (Cu Kα, λ = 1.54 Å).

A Scanning Electron Microscopy (SEM) Quanta 200 FEG (FEI Company, Hillsboro, OR, USA) in environmental mode equipped with an X-Max 80N SDD detector was used to observe the samples and the dispersion of fillers inside the polymer matrix. Voltages of 3 kV for PLA, 10 kV for PUE and fillers were respectively used.

### 2.6. FTIR-ATR Spectroscopy

A FTIR-ATR spectrometer (Vertex 70 FT MIR from Bruker, Billerica, MA, USA) was used to assess the nature of the residue and to determine if the char formed had protected the composite during cone calorimetry testing. The resolution was 4 cm^−1^ and 32 scans for the background and 32 scans for the spectra acquisition were conducted. The spectral range was from 4000 cm^−1^ to 400 cm^−1^ and was analyzed with OPUS software, provided with the spectrometer. The analysis was performed directly on the crystal.

## 3. Results

### 3.1. APP Composites

**Figure 1:**
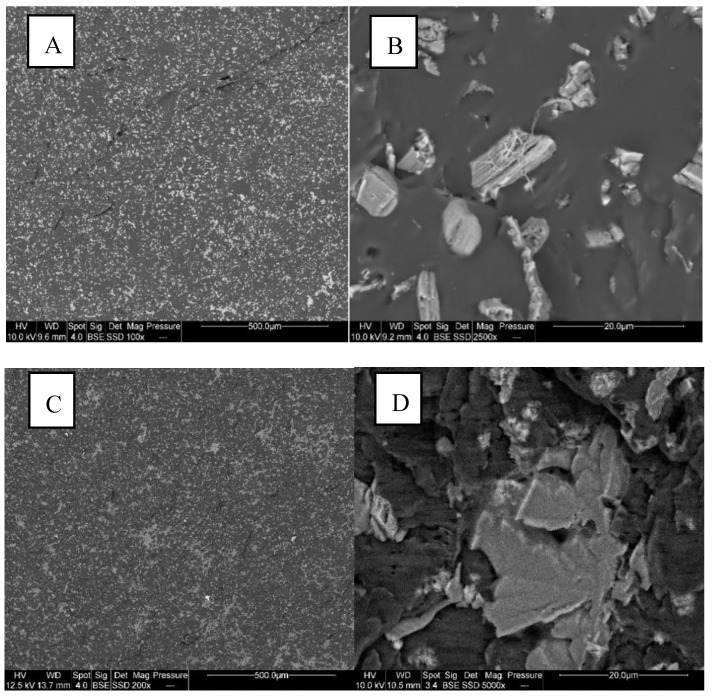
SEM pictures of ammonium polyphosphate (APP)-polyurethane elastomer (PUE) and APP- polylactide (PLA) composites ((**A**) PUE1 ×100; (**B**) PUE1 ×2500; (**C**) PLA1 ×200; (**D**) PLA1 ×5000).

Figure 1 shows the state of dispersion of APP in PUE and PLA. It appears that APP particles are well dispersed into the organic matrices and that the in situ size of particles is the same as the initial one. Even if some aggregates are observed, the blends seem to be homogeneous.

**Figure 2:**
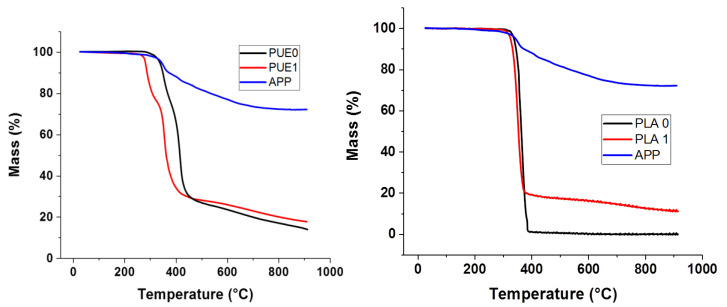
Thermo-Gravimetric Analysis (TGA) for PUE/APP and PLA/APP composites.

Figure 2 and Figure S1 correspond to thermogravimetric and derivative curves of PUE0, PUE1, PLA0 and PLA1. For PUE, two dTG peaks are observed with a main peak at 421 °C. It appears that APP tends to reduce the thermal stability of this polymer. Observing results of TGA in Table 2, T_ON-SET_ of PUE1 is 45 °C lower than PUE0. T_max_ has been also strongly shifted to lower temperatures. However, maximal loss rate is similar between PUE0 and PUE1.

In the same way, the addition of 20% of APP in PLA significantly reduces the maximum mass loss rate (Figure 2 and Figure S1). A small decrease in the maximum degradation temperature and on-set temperature is observed. Moreover, the 12.6% of residue at 900 °C (Table S1) in PLA1 is ascribed to the formation of a compound resulting from the presence of APP.

The difference between the calculated percentage of residue (obtained by using a mixing law of the values of residue of the various components obtained by TGA) and the experimental one is ascribed to the reaction between APP and the polymers and it is higher for PUE in comparison with PLA. Concerning PLA containing APP, the improvement of fire properties seems to be more important. This appears to be more related to the protective structures formed by APP decomposition than to interactions between APP and PLA.

**Figure 3:**
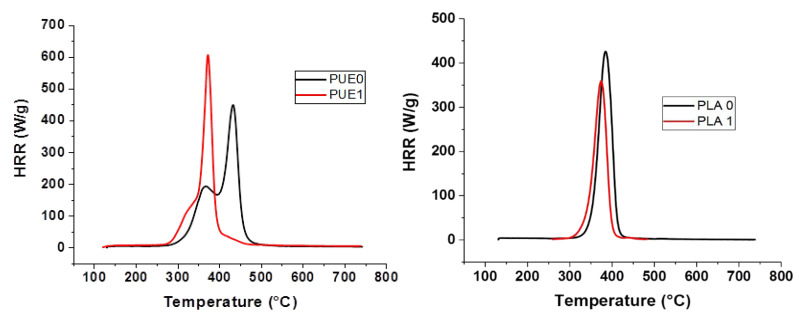
Pyrolysis combustion flow calorimeter (PCFC) curves for PUE/APP and PLA/APP composites.

Investigations of thermal stability were completed by experiments using microcalorimetry of combustion (PCFC). Heat release rate results of PUE0, PUE1, PLA0 and PLA1 are shown in Figure 3 and Table 2.

Results are consistent with the loss of thermal stability in two steps observed using TGA, because the time of pHRR is reduced by 60 °C for PUE1. Moreover, pHRR is 158 W/g higher than for PUE0 due to the very steep temperature ramp and the weight of the sample, the protective effect of a charred structure cannot operate.

PCFC analysis also confirms that the degradation temperature of PLA is slightly shifted in presence of APP. The pHRR of the neat PLA (432 W/g) is decreased of 14.4% by the addition of APP. pHRR temperature is lowered due to the APP degradation. However, it has to be observed that the Total Heat Release (THR) decreased by 21.9% with the addition of APP. This result does not indicate a significant influence on THR since the percentage of flammable material has been reduced by 20% for this composition.

**Figure 4:**
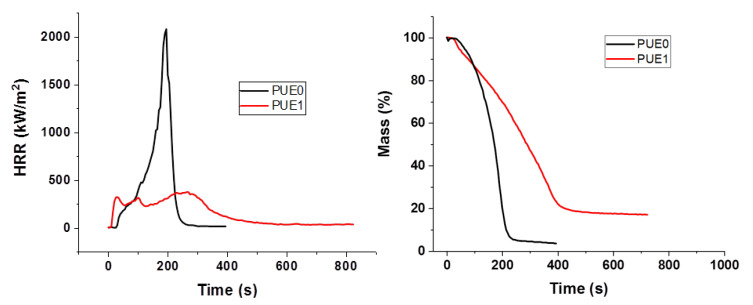
Heat release rate and mass for PUE/APP composites from cone calorimeter.

The fire reaction of materials was studied using a cone calorimeter with an incident heat flux of 50 kW/m^2^. Figure 4 shows the heat release rate (HRR) and mass loss versus time of PUE. Table 3 summarizes the main data obtained for neat materials and APP composites. PUE burns almost completely without charring (only 3.2% of residues). As expected, presence of APP decreases time to ignition (TTi) due to a lower thermal stability than PUE0. However, the peak of HRR (pHRR) is dramatically reduced along with the MARHE. The same tendency is also visible for the mass loss curves. Total Heat Release (THR) of PUE1 is lower than PUE0 due to the effectiveness of APP as flame retardant.

**Figure 5:**
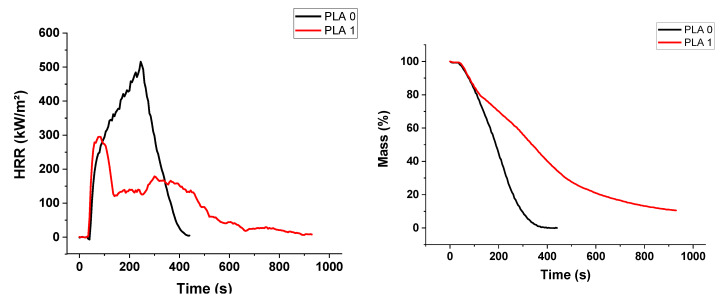
Heat release rate and mass for PLA/APP composites from cone calorimeter.

APP improves also the fire behavior of PLA, as shown in Figure 5. Unlike PUE, a longer time is needed to ignite PLA1. Despite the fact that the TGA and PCFC degradations occur at a lower temperature, pHRR, THR and MARHE are drastically decreased. These results suggest that in PUE, APP sensitizes the thermal degradation in increasing the char and reducing the flammable volatiles.

### 3.2. Combinations of APP with Sepiolite

**Figure 6:**
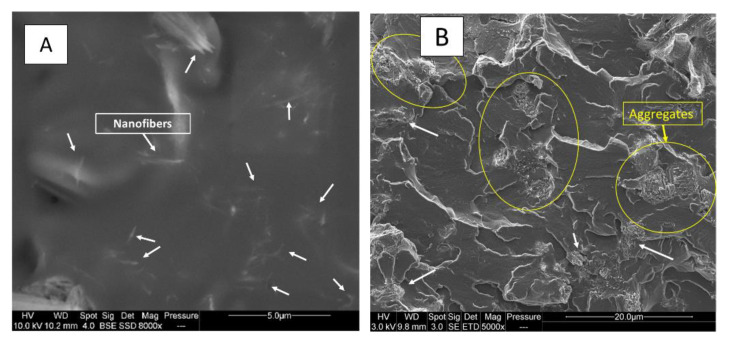
SEM pictures of PUE/APP/S9 and PLA/APP/S9 ((**A**) PUE/APP/S9 ×8000; (**B**) PLA/APP/S9 ×5000).

Sepiolite has been added as a nanofiller into PUE and PLA matrix with APP in view to improve flame retardancy through a barrier effect for mineral filler used alone and the formation of a silicon phosphate for combinations with APP. Figure 6 shows SEM pictures of S9 incorporated into polyurethane and polylactide. Figure 6A shows a good dispersion of nanoparticles appearing as nanofibers inside polyurethane. However, sepiolite inside PLA is less dispersed as nanofibers and forms some aggregates (Figure 6B).

**Figure 7:**
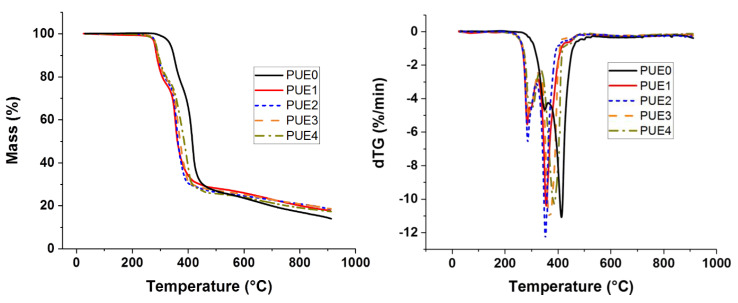
TG and derivates of thermogravimetric curve (DTG) for PUE/APP/S9 composites.

Thermogravimetric curves and derivative curves in Figure 7 show only a slight difference between PUE1 and PUE with S9 (PUE2 to PUE4). In Table S2, T_on-set_ is similar for those formulations. However, an increase in T_Max_ is observed as function of the sepiolite rate in the composite. Maximal mass loss rate is almost the same for all composites. S9 rate seems to have no effect on the mass loss rate and on the rate of degradation.

**Figure 8:**
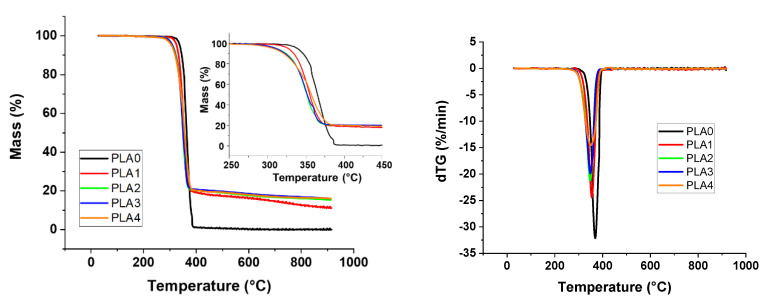
TG and DTG for PLA/APP/S9 composites.

Conversely, the addition of S9 and APP in PLA induces degradation at a lower temperature, as shown in Figure 8. The TG graph shows that there is a decrease of 27 °C for the T_on-set_ with the addition of 3% and 5% of S9 and a drop of 31 °C with the addition of 8% of S9. Maximum degradation temperatures are quite identical for all the compounds and the decomposition occurs always in two steps. Conversely, the mass loss is increased in comparison with all singular materials. Moreover, the addition of S9 leads to an experimental value of residue that is higher than the theoretical one.

**Figure 9:**
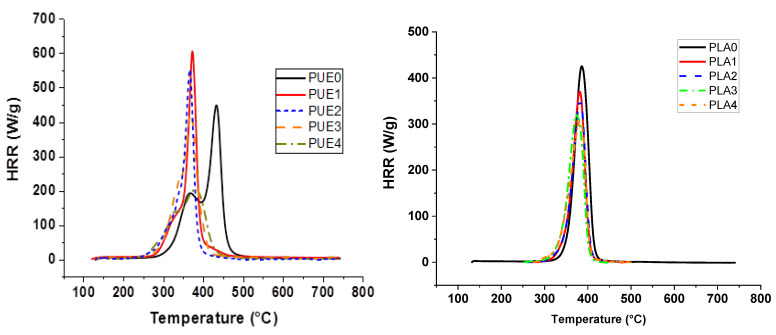
PCFC curves for PUE/APP/S9 and PLA/APP/S9 composites.

PCFC results highlight a possible barrier effect imparted by the combination of APP and sepiolite since pHRR is reduced for both polymers (Table 4). In addition, the greater the sepiolite ratio, the greater the reduction of pHRR for PUE compositions, as shown in Figure 9. The effect is particularly significant in the case of PUE4 for which it can also be observed that THR was also strongly reduced.

For PLA compositions, the behavior as function of the percentage of sepiolite is more progressive. A continuous decrease in pHRR can be noticed as function of S9, but there is no influence on THR.

**Figure 10:**
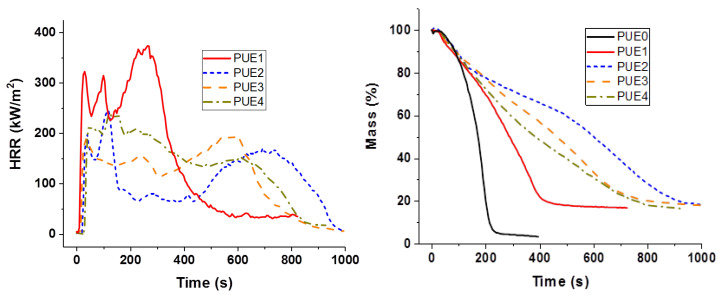
Heat release rate and mass for PUE/APP/S9 composites from cone calorimeter.

The increase in the S9/APP ratio leads to significant differences for the cone calorimeter tests between PUE and PLA. For PUE in Figure 10, a synergistic effect between APP and sepiolite can be observed with regard to HRR vs. time profiles and derived fire parameters listed in Table 5. This can be noted particularly for PUE2 and PUE3 since the replacement of, respectively, 3 and 5 wt% of APP by sepiolite leads to a significant decrease in pHRR, THR and MAHRE in comparison with PUE1 which contains only APP. A synergistic effect is also noticed for a higher substitution of APP in PUE4 (8 wt%) but only for pHRR and MAHRE. A percentage of 5% of sepiolite corresponds to the optimum of performance for all fire reaction parameters. In particular, the final residue is maximized (20.9%).

**Figure 11:**
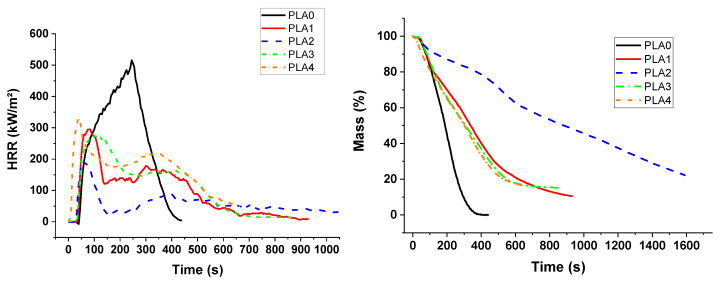
Heat release rate and mass for PLA/APP/S9 composites from cone calorimeter.

For PLA materials, cone calorimeter measurements (Figure 11) show that there is also an optimum of performance for the APP/S9 ratio. The PLA 2 formulation with 3 wt% of sepiolite leads to the highest fire performance. The width of the HRR peak is less for the most interesting composite formulations and the peaks are significantly smaller. The formation of a protective char during the thermal degradation is highlighted regarding the very low values of HRR and the levelling-off of the corresponding curve. The pHRR decreased by 37% (Table 5) and THR also decreased by 63.5% in comparison with neat PLA.

### 3.3. Combinations of APP with Lignin

**Figure 12:**
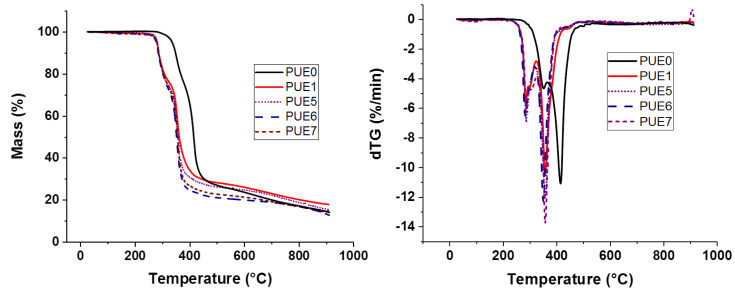
TG and DTG curves for PUE/APP/lignin composites.

Figure S2 and Figure 12 present, respectively, the TG and dTG curves for lignin and PUE/APP/lignin composites. Firstly, the T_ON-SET_ and the T_Max_ of PUE1, PUE5, PUE6 and PUE7 are quite similar, and then the addition of lignin has no real impact on the thermal stability of PUE (Table S3). The maximal loss rate of each composite is also similar to the maximal loss rate for neat PUE. Differences can be noted for the mass of residue, which is not enhanced by the incorporation of lignin. But it can be explained by the decrease in the rate of APP. However, it has to be noticed that the experimental residue is, in all cases, much lower than the theoretical one. Hence, a charring effect of lignin in PUE cannot be established.

**Figure 13:**
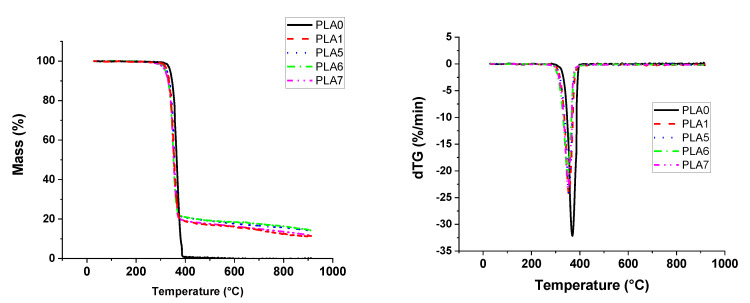
TG and dTG curves for PLA/APP/lignin composites.

Composites with PLA containing APP and lignin present similar TGA curves, whatever the APP/lignin ratio (Figure 13). Lignin degradation is not discernable in TGA and dTG graphs. There is no significant dTG variation from lignin addition between 200 and 400 °C. A slight decrease in T_ON-SET_ is observed, nevertheless the addition of lignin reduces significantly T_MAX_ and the maximal mass loss rate (Table S3).

**Figure 14:**
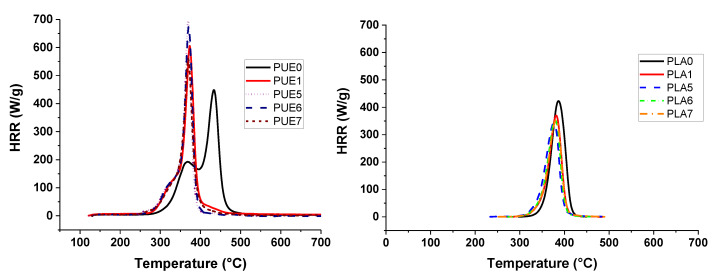
PCFC curves for PUE and PLA/APP/lignin.

PCFC analyses in Figure 14 and Table 6 show an increase in HRR in the presence of lignin for PUE compositions, except for PUE7, even in comparison with samples containing only APP. Moreover, the pHRR temperature is strongly decreased for all flame retarded samples which results from the important interactions between all components during their thermal degradation. Consequently, flame retarded PUE seems to degrade in only one step conversely to neat PUE. For all samples with flame retardants, APP is the major component modifying the degradation of PUE and the lignin has a negligible effect on either the APP or PUE degradation. This is in accordance with the TGA experiments.

Pyrolysis combustion flow calorimetry of PLA-based composites is influenced by the lignin incorporation since the pHRR is lower with the incorporation of lignin (Figure 14). All PLA mixed compositions exhibit lower pHRR than pure PLA and PLA with APP, particularly PLA5. Hence, a synergistic effect between APP and lignin can be then highlighted for this property. Moreover, it can be noted that TGA residue results in Table S3 also suggest a synergistic effect regarding char formation.

As for PUE, all flame retarded compositions show lower THR than pure polymer. Nevertheless, there is no dependence on the presence of lignin.

**Figure 15:**
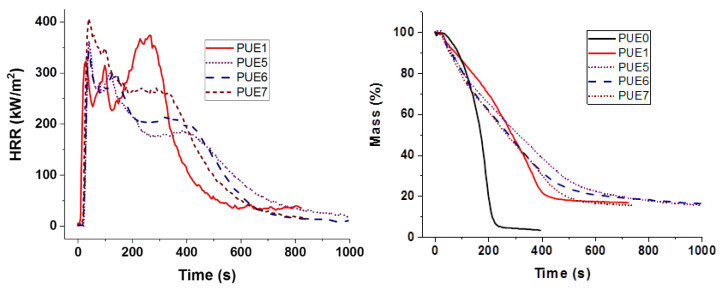
Heat release rate and mass for PUE/APP/lignin composites from cone calorimeter.

Regarding the cone calorimeter experiments, HRR curves for PUE mixed compositions in Figure 15 exhibit higher values of pHRR in comparison to PUE/APP compositions, but the time corresponding to the peak is shorter. From Table 7, THR is only reduced for PUE5 composition which also presents a lower MARHE value than the composition with APP alone.

**Figure 16:**
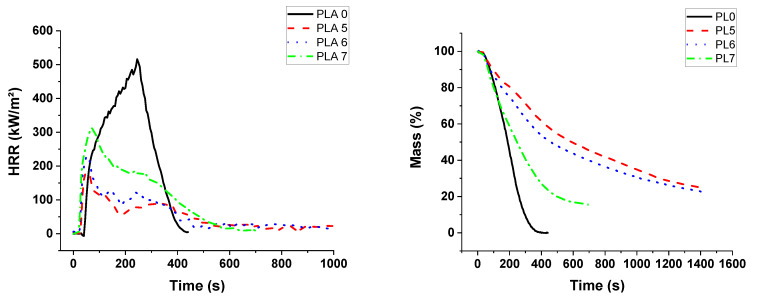
Heat release rate and mass for PLA/APP/lignin composites from cone calorimeter.

Conversely, for PLA, cone calorimeter tests (Figure 16) show that the lignin improves significantly the fire retardancy. pHRR is lower for APP/lignin except for the highest lignin content. Evolutions of THR, MARHE and experimental residues are in accordance with pHRR values, evidencing a synergistic effect between APP and lignin up to 5 wt% lignin in the composition (Table 7). The strong char promoter activity of lignin is highlighted for PLA5 and PLA6. The important reactivity between the components leads to a dramatic reduction in time to ignition.

## 4. Discussion

### 4.1. Compositions with Only APP

**Figure 17:**
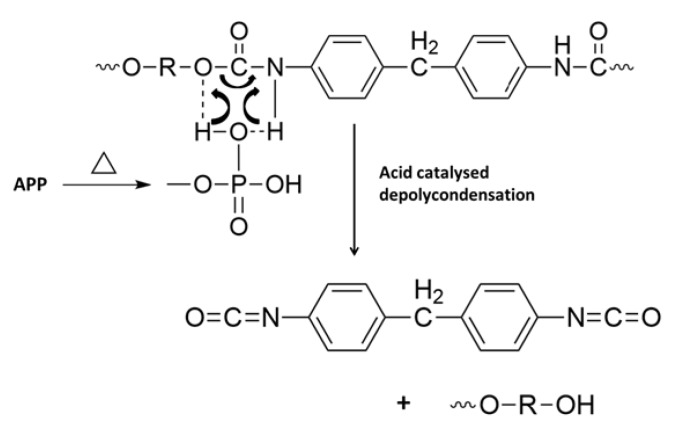
Mechanism of PUE depolymerization in the presence of APP, adapted from Duquesne et al. [65].

TGA curves and PCFC results highlight a stronger reactivity between APP and PUE in comparison with PLA. APP is known to promote charring for PUE ([11,31,35,64]). Indeed, phosphoric acid reacts with hydroxyl groups to lead to an unsaturated and charred structure according to Figure S3. Nevertheless, a dramatic mass loss of PUE is observed. It is due to an important release of degradation products with an experimental residue scarcely higher than that obtained with pure PUE. This phenomenon can be ascribed to the influence of APP which catalyzes polyurethane de-polymerization, this effect being described in the literature ([38,65]). Indeed, de-polymerization is the main mechanism of thermal degradation for PUE. For PUE1, polyphosphoric acid formed from APP thermal decomposition leads to an acid catalyzed de-polycondensation of polyurethanes to alcohol and isocyanate groups, as shown in Figure 17 ([33,66]). Unlike PUE, PLA does not char and so the APP mode of action is limited if there is no incorporation of a strong char promoter required to form an intumescent fire-retardant system. Then, in this case, an interaction and even synergy between APP and lignin could be expected with lignin playing a role of charring agent [62,67,68].

Despite the loss of thermal stability due to the presence of APP, the evolution of HRR as a function of time measured by cone calorimetry decreases more strongly for PUE than for PLA. For the former, the pHRR reduction is 82% whereas only 43% is noted for the latter. The formation of the charred structure appears very quickly after the ignition for PUE1, even if the HRR curve is not completely flattened, showing a limited stability of the charred structure formed. Conversely for PLA, during the first hundred seconds, the behavior of PLA1 is similar to pure PLA, which indicates a limited ability to char quickly. Nevertheless, the appearance of a plateau highlights the formation of a protective structure, corresponding to a thick charring behavior ([69]). The higher effectiveness of APP in PUE can also be noticed by considering the aspect of residues (Figure S4). The amount of char in PUE1 is higher, and more expanded than the amount of char for PLA1, and seems more cohesive. XRD characterizations on PUE and PLA cone calorimeter residues shown in Figure S5 reveal the presence of a poor crystallized structure, particularly for PUE1, but show some differences in the charred structures.

### 4.2. Compositions with APP and Sepiolite

It has been noticed that the incorporation of sepiolite in combination with APP results in opposite effects on the thermal stability of PUE and PLA. For PUE, the increase in Tmax can be ascribed on one hand to a reduced effectiveness of APP which may be due to the formation of new chemical species such as silicon phosphate, already observed in previous works [46,58]. The reduction in the difference between the experimental and theoretical residue seems to support this last interpretation.

The contribution of sepiolite to improve the fire behavior is significant for all PUE compositions with an optimum APP/Sepiolite ratio of 3 (PUE 3) from cone calorimeter tests. It can be considered that the formation of new phosphates acting as a ceramic protective layer occurs very quickly for the rapid temperature rises involved in PCFC and cone calorimeter tests [46,58]. In addition, it can also be suggested that the presence of free sepiolite could also impart a barrier effect entailing an increase in thermal stability and fire performance.

**Figure 18:**
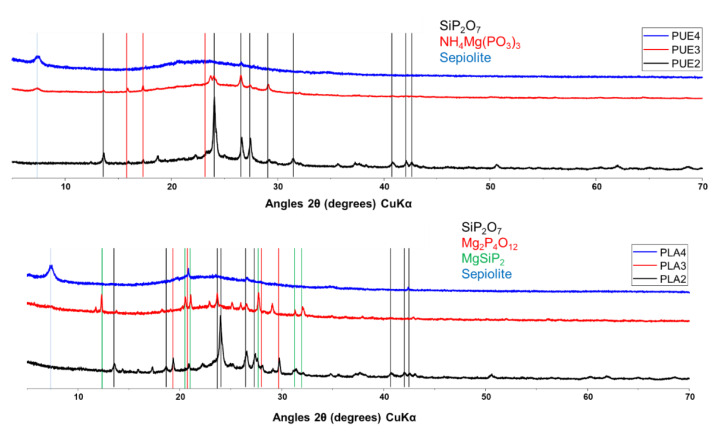
XRD patterns for PLA/APP/S9 and PUE/APP/S9 composites residues. (The colors of the vertical lines are in agreement with those of the compounds identified.)

XRD analyses carried out on cone calorimeter residues presented in Figure 18 show the formation of silicon and magnesium phosphorus compounds as well as the presence of free sepiolite dependent on the APP/sepiolite ratio. Since the fire performance is ranked according to: PUE 3 > PUE 2 > PUE 4, it can be concluded that it seems more advantageous to select an intermediate ratio allowing for both phosphorus compounds (SiP_2_O_7_ and NH_4_Mg(PO_3_)_3_) to be formed but also to have a remaining fraction of sepiolite which could reinforce this protective ceramic and charred structure [70].

The influence of the incorporation of sepiolite is less effective for PLA. Regarding thermal stability, the levelling off of Tmax as a function of the sepiolite rate seems to indicate that sepiolite could cause hydrolytic degradation of PLA due to water release and de-hydroxylation. This phenomenon could be able to offset positive effects such as restrictions of molecular chains entailing a barrier effect and formation of a protective layer owing to the formation of new phosphorus species. Moreover, aggregation phenomena of the nanoparticles for percentages higher than 3 wt% could also limit the interest in their incorporation.

Nevertheless, pHRR values are reduced during PCFC testing for all mixed compositions in comparison with APP alone, but conversely this improvement of fire behavior is not observed for all compositions during cone calorimeter tests. Only PLA 2 with the highest APP/sepiolite ratio of 5.67 shows an improvement of all fire parameters in comparison with APP alone. In addition, as it can be seen in Figure S6, residues of PLA composites are only slightly intumescent, unlike those from PUE composites, therefore suggesting that different mechanisms occur to account for respective fire performances. The examination of XRD spectra shows that for PLA 2, the formation of phosphorus compounds appears to be more clearly evident whereas no free sepiolite can be identified. Hence, it can be assumed that in this case, the detrimental effect of sepiolite on PLA hydrolysis could be limited to the rapid consumption of the mineral to form the protective layer made of the new phosphorus species.

### 4.3. Compositions with APP and Lignin

Lignin has been shown to be a charring agent [36,37,56]. During the thermal degradation of lignin various reactions occur to form a charred structure which is expected to protect the polymers in which lignin is incorporated. The decompositions range occurs between 280 and 365 °C and corresponds to de-alkylation, de-methylation and carbonization reactions [71]. The TGA curve of lignin (Figure S2) shows the dehydration step of the sample around 100 °C. Between 200 and 400 °C, lignin degradation occurs with different decomposition and re-polymerization stages. From 800 °C, degradation of the lignin carbon backbone can be seen [72].

For the PUE composites, the difference between the experimental residue and the calculated one could be ascribed to an additional catalysis effect on PUE degradation by lignin acidic functions, leading to more de-polycondensation. Hence, degradation products issued from PUE become shorter and their release rate is more important as seen also in PCFC tests. This phenomenon of de-polycondensation has been previously reported by Zhang et al. [73]. Tetrahydrofuran (THF), water CO and CO_2_ have been identified as the main products of the thermal degradation of PUE. Hence, the presence of lignin does not entail a real improvement in PUE fire retardancy, unlike sepiolite which exhibits better performance for the same ratio.

It may be suggested that for a partial substitution of APP by lignin, the fire performance could be maintained for a substituted amount of APP of about 25% of the maximum, as in the cases of PUE5 and PUE6. In addition, the intumescent character is not significant for PUE containing lignin, the converse of mixed PLA compositions as shown in Figure S7.

For PLA compositions, it seems that the various amounts of lignin and APP in PLA have no influence on the maximum degradation temperature. However, lignin mixed with APP leads to a higher experimental mass residue than the theoretical one, compared to APP alone. This results from the charring effect promoted by lignin and it proves the existence of interactions between all blend components during their thermal degradation.

From a cone calorimeter, synergistic effects on fire performance were observed for 3 and 5 wt% of lignin, with a significant increase in residue, but not for 8 wt%. It can be suggested that the charring effect was limited to a certain APP/lignin ratio (possibly close to three). Beyond this value, the amount of APP is considered as too low for such a positive interaction to occur. The char formed after the degradation protects the materials since the HRR decreases continuously after the pHRR.

Char residues were analyzed by Infrared spectroscopy in order to determine the chemical structure of residue underneath the charred layer, in comparison with the one of the initial polymer (Figure S8). An expanded crust was formed during cone calorimetry testing of PLA6 and this crust was not fractured during the test. For the other formulation the crust was fractured and all the samples were totally burnt. The IR spectra for PLA6 and its residue under the crust revealed that the polymer was fully protected by the charred structure. The spectrum seems to be identical. An increase in the linked OH group observed between 3660 and 3400 cm^−1^ could be attributed to the oxidation of the polymer matrix and/or the detection of lignin. In addition, the residue spectrum exhibits a peak at 974 cm^−1^ that can be related to the P-O-C bond stretch, resulting from a reaction between APP and lignin. All the cone calorimeter residues of the composites containing lignin were analyzed by XRD. However, all results showed only amorphous phases. No information was obtained on the possible formation of crystallized phosphorus compounds during the thermal degradation of these composites.

## 5. Conclusions

All the flame retardants systems based on APP combined with sepiolite or lignin allowed the fire performance of PUE and PLA to be improved or at least maintained. The use of APP alone led to an improvement of fire parameters in terms of reduced pHRR, THR and MARHE and mass loss rate. However, the action of APP appeared different for both polymers. TGA and PCFC results clearly showed that in the case of PUE, the thermal stability was more strongly impaired than in the case of PLA, in order to promote a charred and intumescent structure. Moreover, for PUE, PCFC showed that the acid catalyzed de-polycondensation caused by APP entails a dramatic release of energy at a lower temperature than was noted for PUE alone.

In the cone calorimeter, the reduction in time to ignition for PUE was also due to the detrimental effect of APP on thermal stability in order to promote a charred and intumescent structure, which was not so significant in the case of PLA. Hence, the decrease in pHRR for PUE was more important (82%) than for PLA (43%) for which no significant intumescent structure was found.

Sepiolite was introduced in order to create a barrier effect through a direct reinforcement of the char or through the formation of silicon phosphate and other phosphorus compounds by reaction with APP. A synergistic effect on the fire parameters in the cone calorimeter was noticed for all compositions with PUE. An optimum APP/sepiolite ratio of three was highlighted and it can be concluded that there was no competition between the formation of the intumescent structure from PUE and APP and the formation of silicon phosphate and other phosphorus components from sepiolite and APP. For PLA, the synergistic effect was also highlighted but only for one composition and a higher APP/sepiolite mass ratio (close to six). From TGA curves, it can be noted that sepiolite, for which a barrier effect was expected due to possible restrictions of macromolecular chains, led in fact to a reduction of thermal stability. It can be ascribed to water release and de-hydroxylation of hydroxyl groups leading to PLA hydrolysis. Hence, the interest of sepiolite towards formation of protective structure through silicon phosphate or other phosphorus compounds formation was here limited due to a detrimental effect on thermal stability.

The combination of lignin with APP was expected to impart a stronger charring effect. In the case of PLA, a reduction in pHRR was noticed in PCFC testing without a significant reduction in time for the peak value. From cone calorimetry, synergistic effects on fire performance were observed for an APP/lignin ratio up to three. Conversely, for PUE no synergistic effect was noticed and the amount of residue formed during cone calorimetry tests was slightly reduced. This lack of performance of lignin in PUE can be explained by an increased catalytic effect on de-polycondensation since PCFC tests showed a strong increase in pHRR at the same temperature as PUE with APP alone. Hence, the char promoting effect of lignin was completely offset by its detrimental effect on the thermal stability of PUE, resulting in a strong release of volatile combustibles during the cone calorimeter test.

## Figures and Tables

**Table 1:** List of compositions.

Polymer Matrix	APP [wt%]	Sepiolite [wt%]	Lignin [wt%]
PUE 0	-	-	-
PUE 1	20	-	-
PUE 2	17	3	-
PUE 3	15	5	-
PUE 4	12	8	-
PUE 5	17	-	3
PUE 6	15	-	5
PUE 7	12	-	8
PLA 0	-	-	-
PLA 1	20	-	-
PLA 2	17	3	-
PLA 3	15	5	-
PLA 4	12	8	-
PLA 5	17	-	3
PLA 6	15	-	5
PLA 7	12	-	8

**Table 2:** PCFC results for PUE/APP and PLA/APP composites.

Polymer Matrix	Phrr (W/g)	T of pHRR (°C)	THR (kJ/g)
PUE0	448	433	25.6
PUE1	606	373	23.7
PLA0	432	387	17.8
PLA1	370	381	13.9

**Table 3:** Cone calorimeter results for PUE/APP and PLA/APP composites.

Polymer Matrix	TTi (s)	pHRR (kW/m^2^)	THR (MJ/m^2^)	MARHE (kW/m^2^)	Experimental Residue (%)
PUE0	22	2080	147	628	3.2
PUE1	12	373	118	274	16.3
PLA0	33	516	108	311	0
PLA1	49	295	87	172	10.5

**Table 4:** PCFC results for PUE/APP and PLA/APP composites.

Polymer Matrix	pHRR (W/g)	T of pHRR (°C)	THR (kJ/g)
PUE0	448	433	25.6
PUE1	606	373	23.7
PUE2	553	367	22.5
PUE3	426	369	23.9
PUE4	207	382	19.6
PLA0	432	387	17.8
PLA1	370	381	13.9
PLA2	351	382	14.2
PLA3	322	375	14.4
PLA4	307	383	14.1

**Table 5:** Cone calorimeter results for PUE/APP/S9 and PLA/APP/S9 composites.

Polymer Matrix	TTi (s)	pHRR (kW/m^2^)	THR (MJ/m^2^)	MAHRE (kW/m^2^)	Experimental Residue (%)	Theorical Residue (%)
PUE0	22	2080	147	628	3.2	13.7
PUE1	12	373	118	274	16.3	28.1
PUE2	19	245	107	152	17.6	25.7
PUE3	16	194	110	148	20.9	26.0
PUE4	21	240	126	184	16.2	26.3
PLA0	33	516	108	311	0	0
PLA1	49	295	87	172	10.5	14.4
PLA2	30	192	69	109	17.2	14.8
PLA3	33	281	97	192	14.9	15.1
PLA4	11	331	109	226	16.3	15.4

**Table 6:** PCFC results for PUE and PLA/APP composites.

Polymer Matrix	pHRR (W/g)	T of pHRR (°C)	THR (kJ/g)
PUE0	448	433	25.6
PUE1	606	373	23.7
PUE5	695	369	22.5
PUE6	674	371	23.4
PUE7	560	370	23.1
PLA0	432	387	17.8
PLA1	370	381	13.9
PLA 5	341	375	14.3
PLA 6	355	378	13.8
PLA 7	353	381	14

**Table 7:** Cone calorimeter results for PUE and PLA/APP/lignin composites.

Polymer Matrix	TTi (s)	pHRR (kW/m^2^)	THR (MJ/m^2^)	MARHE (kW/m^2^)	Experimental Residue (%)	Theorical Residue (%)
PUE0	22	2080	147	628	3.2	13.7
PUE1	12	373	118	274	16.3	28.1
PUE5	16	378	109	217	15.4	24.6
PUE6	16	341	124	238	15.1	24.1
PUE7	17	371	132	247	15.7	23.3
PLA0	33	516	108	311	0	0
PLA1	49	295	87	172	10.5	14.4
PLA5	29	180	56	104	24.8	13.6
PLA6	26	229	62	129	22.0	13.0
PLA7	23	312	82	209	15.4	12.2

## Supplementary Materials

The following are available online at https://www.mdpi.com/1996-1944/13/11/2450/s1, Figure S1: DTG for PUE/APP and PLA/APP composites, Figure S2: TG and dTG curves of lignin, Figure S3: Mechanism of char formation, Figure S4: Residue of cone calorimeter test of PLA1 and PUE1, Figure S5: XRD patterns for PLA/APP and PUE/APP composites residues, Figure S6: Residues of cone calorimeter test of APP/S9 series, Figure S7: Residues of cone calorimeter testing of APP/lignin series, Figure S8: IR spectrum for PLA6 and PLA6 residue, Table S1: TGA results for PLA and PUE composites filled with APP, Table S2: TGA results for PUE/APP/S9 and PLA/APP/S9 composites, Table S3: TGA results for PUE/APP/LIGNIN and PLA/APP/lignin composites.
