# Supplementary material for: Influence of Sepiolite and Lignin as Potential Synergists on Flame Retardant Systems in Polylactide (PLA) and Polyurethane Elastomer (PUE)
Source: Materials (Basel). 2020 May 28;13(11):2450. doi: 10.3390/ma13112450 (PMC7321226; doi:10.3390/ma13112450)
Supplement: Supplementary file 1 [file materials-13-02450-s001.pdf]

materials

MDPI

Supplementary Part

Influence of Sepiolite and Lignin as Potential Synergists on Flame Retardant Systems in Polylactide (PLA) and Polyurethane Elastomer (PUE)

Valentin Carretier, Julien Delcroix, Monica Francesca Pucci, Pierre Rublon and José-Marie Lopez-Cuesta

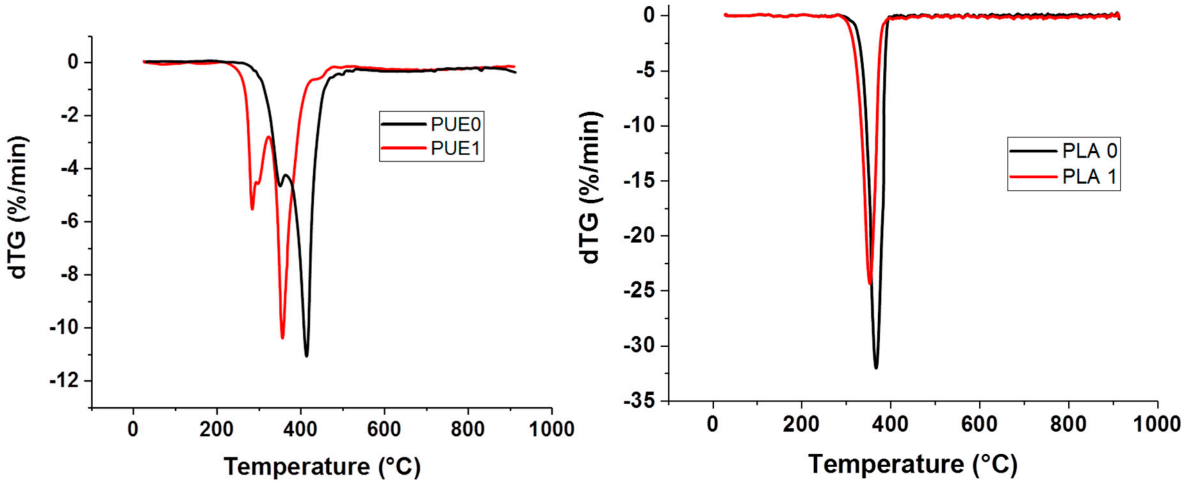

Figure S1. DTG for PUE/APP and PLA/APP composites.

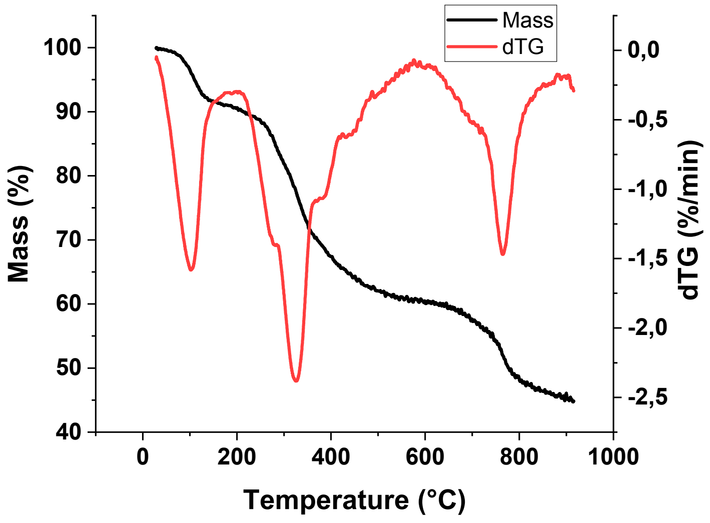

Figure S2. TG and dTG curves of lignin.

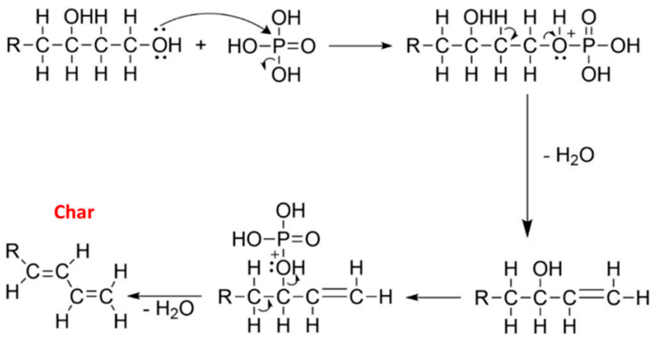

Figure S3. Mechanism of char formation.

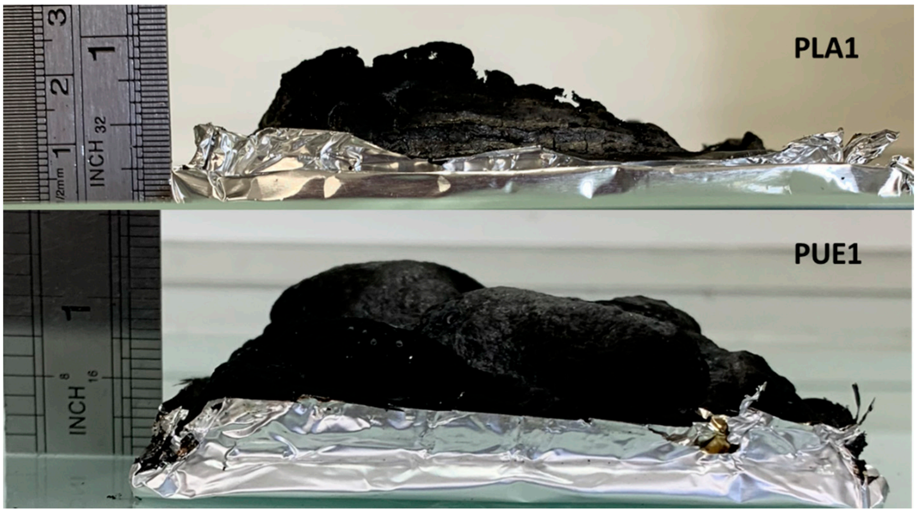

Figure S4. Residue of cone calorimeter test of PLA1 and PUE1.

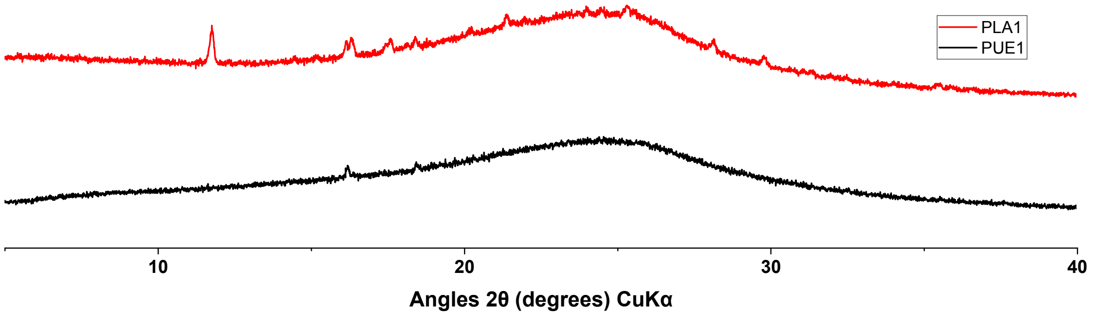

Figure S5. XRD patterns for PLA/APP and PUE/APP composites residues.

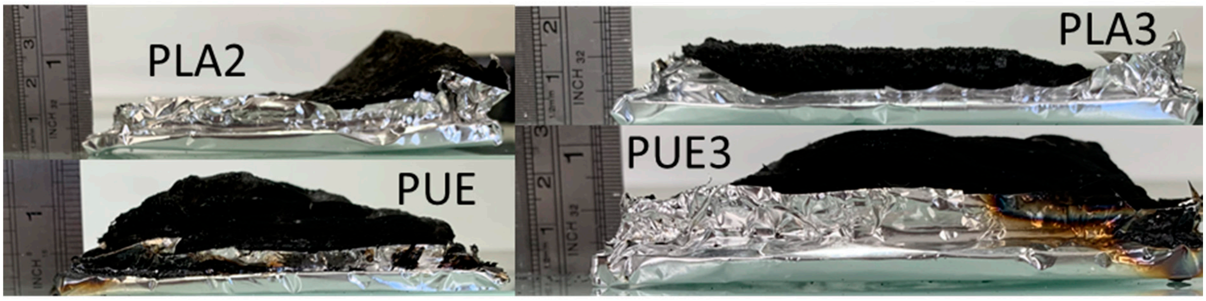

Figure S6. Residues of cone calorimeter test of APP/S9 series.

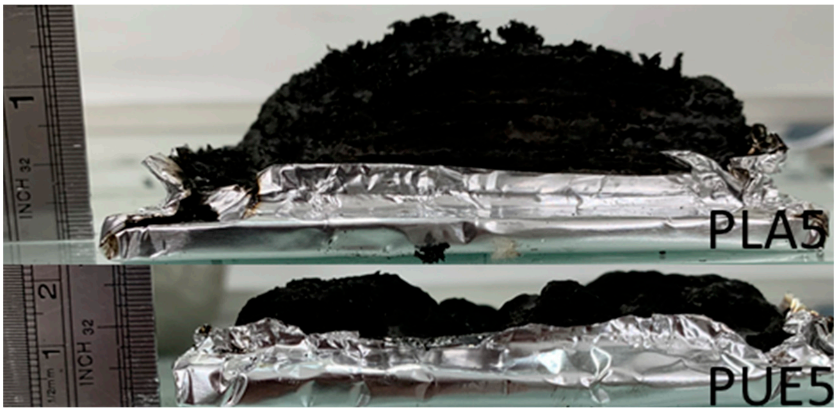

Figure S7. Residues of cone calorimeter testing of APP/lignin series.

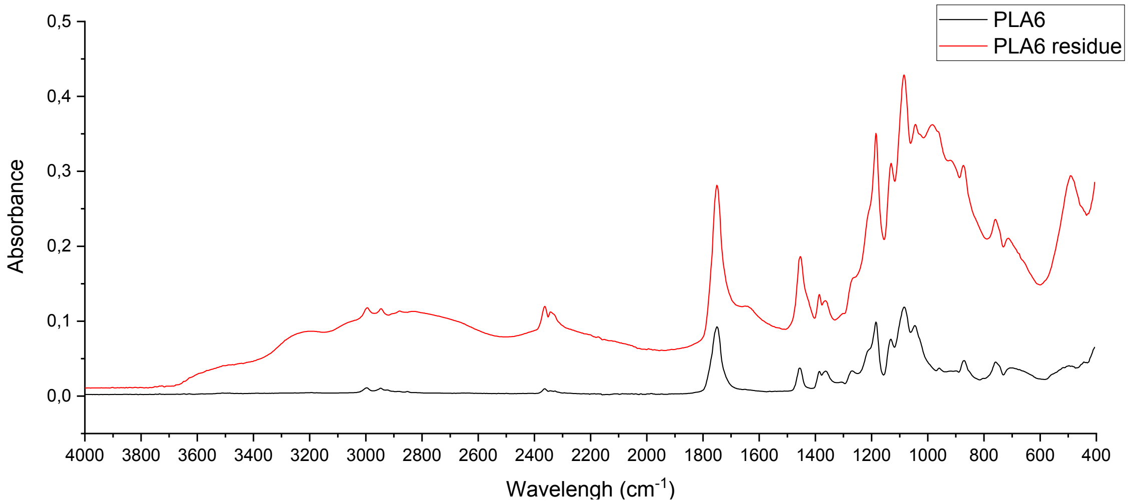

Figure S8. IR spectrum for PLA6 and PLA6 residue.

Table S1. TGA results for PLA and PUE composites filled with APP.

	$T_{ON\text{-}SET}$ (°C)	T Max (°C)	Maximal mass loss rate (%/min)	Experimental Residue (%) (900°C)	Calculated residue (%)
**APP**	344	363	-2	72.0	72.0
**PUE0**	331	421	-10	13.7	13.7
**PUE1**	286	358	-10	17.5	28.1
**PLA 0**	338	367	-32	0	0
**PLA 1**	326	355	-24	11.3	14.4

Table S2. TGA results for PUE/APP/S9 and PLA/APP/S9 composites.

	$T_{ON\text{-}SET}$ (°C)	T Max (°C)	Maximal mass loss rate (%/min)	Residue (%) (900°C)	Calculated residue (%)
**APP**	344	363	-2	72.0	-
**S9**	100	294	-0.31	83.8	-
**PUE0**	331	421	-10	13.7	-
**PUE1**	286	358	-10	17.5	28.1
**PUE2**	285	362	-12	20.3	25.7
**PUE3**	286	372	-11	20.2	26.0
**PUE4**	286	387	-10	18.5	26.3
**PLA0**	338	367	-32	0	-
**PLA1**	326	355	-24	12.6	14.4
**PLA2**	311	347	-21	15.6	14.8
**PLA3**	310	349	-20	16.4	15.1
**PLA4**	307	352	-15	16.1	15.4

Table S3. TGA results for PUE/APP/LIGNIN and PLA/APP/lignin composites.

	$T_{ON\text{-}SET}$ (°C)	T Max (°C)	Maximal mass loss rate (%/min)	Residue (%) (900°C)	Calculated residue (%)
**APP**	344	363	-2	72.0	-
**Lignin**	110	327	-2.4	44.6	-
**PUE0**	331	421	-10	13.7	-
**PUE1**	286	358	-10	17.5	28.1
**PUE5**	281	356	-12	15.4	24.6
**PUE6**	280	356	-12	12.2	24.1
**PUE7**	282	358	-14	14.4	23.3
**PLA0**	338	367	-32	0	-
**PLA1**	326	355	-24	11.3	14.4
**PLA5**	326	355	-24	15.6	13.6
**PLA6**	319	351	-21	14.2	13.0
**PLA7**	319	351	-23	13.6	12.2
